# Germ cell tumors, cell surface markers, and the early search for human pluripotent stem cells

**DOI:** 10.1002/bies.202400094

**Published:** 2024-08-08

**Authors:** Peter W. Andrews

**Affiliations:** ^1^ The Centre for Stem Cell Biology The School of Biosciences The University of Sheffield Western Bank Sheffield UK

**Keywords:** cell surface antigens, embryonal carcinoma cell, human, pluripotent stem cell, teratocarcinoma

## Abstract

Many strands of research by different groups, starting from teratocarcinomas in the laboratory mouse, later moving the corresponding human tumors, contributed to the isolation and description of human pluripotent stem cells (PSCs). In this review, I highlight the contributions from my own research, particularly at the Wistar Institute during the 1980s, when with my colleagues we characterized one of the first clonal lines of pluripotent human embryonal carcinoma (EC) cells, the stem cells of teratocarcinomas, and identified key features including cell surface antigen markers that have since found a place in the study and exploitation of human PSC. Much of this research depended upon close teamwork with colleagues, many in other laboratories, who contributed different expertise and experience. It was also often driven by circumstance and chance rather than pursuit of a grand design.

## 1 INTRODUCTION

My introduction to human embryonal carcinoma (EC) cells in1978 was coincidental. After a PhD from Oxford University, which gave me a background in *Drosophila* developmental genetics my first two postdoc positions, in the groups of François Jacob at the Institut Pasteur in Paris and Ted Boyse at the Sloan Kettering Institute in New York, focused on the potential of mouse teratocarcinomas (Box 1) and their EC stem cells as tools for dissecting mouse embryology, and on cell surface antigens as tools for following cell differentiation. Some of my work at the Pasteur focused on “the F9 antigen”, defined by the reactivity of serum from syngeneic mice immunized with the mouse teratocarcinoma cell line, F9. This F9 antigen was generally detectable on mouse EC cell lines and preimplantation mouse embryos, and was initially thought to be encoded by the mouse T‐locus, itself suggested to regulate early embryogenesis.^[^ ^1^, ^2^ ^]^ As discussed by Robert Erickson, elsewhere in this issue,^[^ ^3^ ^]^ there were serious flaws in this idea and so, after nearly 4 years of postdoc research, I had published little and had yet to settle on a distinct project.

Nevertheless, while in New York, I had taken several vacations to visit a friend, Peter Goodfellow, who had been a graduate student with me at Oxford and was then a post doc with Hugh McDevitt at Stanford University. During those trips, we had used somatic cell hybridization techniques to explore the control of pluripotency in mouse EC cells^[^ ^4^ ^]^ and developed ideas for searching for new differentiation antigens to dissect the pathways of differentiation from mouse EC cells.

I was then fortunate to contact the Barbara Knowles and Davor Solter at the Wistar Institute (Box 2). From my previous work, I had some experience relevant to their interests as they had recently described a monoclonal antibody that recognized a mouse preimplantation stage‐specific antigen (SSEA1), also expressed on the immunizing F9 EC cells.^[^ ^5^ ^]^ Moreover, with the developing use of mouse teratocarcinomas as models for early mouse embryos, as described by Papaioannou in this issue,^[^ ^6^ ^]^ Barbara and Davor had been exploring the possibility of using cell lines from human germ cell tumors, which include teratocarcinomas, as tools for studying human embryogenesis. To this end they had recently acquired a panel of cell lines derived from clinical samples of human testicular germ cell tumors in the University of Minnesota, one of the main centers for treating this type of cancer. Up till then, the principal interest in these tumors was from clinicians and oncologists. Although rare overall, testicular germ cell tumors are most common in young men and survival rates were very poor until the inclusion of *cis*‐platinum into therapy dramatically changed the outcomes.^[^ ^7^ ^]^ We all decided our experience and interests overlapped sufficiently, so I moved to Wistar to delve more into characterizing the structure of the SSEA1 antigenic determinant^[^ ^8^ ^]^ and to try to identify undifferentiated EC cells in the germ cell tumor cell lines, and find evidence that they differentiated in culture. There was a link to SSEA1, since two papers had been published showing that the molecularly undefined murine “F9 antigen” could be serologically detected in cultures of human teratocarcinomas and inferring that *human* EC cells were also marked by the “F9 antigen”.^[^ ^9^, ^10^ ^]^ I therefore started our work on the human lines assuming that human EC cells would also express SSEA1. Indeed we found that most of the human teratocarcinoma cell lines to which we had access did contain SSEA1‐positive cells, but the story proved more complex.

**Teratoma and Teratocarcinoma Terminology**

The term “Teratoma” was coined by Robert Virschow in 1869^[^ ^11^ ^]^ to describe tumors comprising multiple tissues that typically, though not exclusively, occur in the testes and ovaries. In humans, ovarian teratomas are usually benign but testicular teratomas are almost invariably malignant. Friedman, in 1946,^[^ ^12^ ^]^ proposed the term teratocarcinoma to describe the testicular tumors, the malignancy of which he ascribed to the presence of undifferentiated cells, embryonal carcinoma (EC) cells, that could differentiate to form the other differentiated cells of the tumor. This was clearly demonstrated in the case of the experimental teratomas of the laboratory mouse, notably by the study of Kleinsmith and Pierce,^[^ ^13^ ^]^ who showed that single EC cells are indeed able to initiate the development of a tumor that contains mature somatic tissues. It then became well established terminology among researchers studying the laboratory mouse, to use the term “teratoma” to denote a tumor composed of mature somatic tissues corresponding to all three germ layers, and the term “teratocarcinoma” to denote such a tumor that also contained EC cells.^[^ ^14^, ^15^ ^]^ Nevertheless, diagnostic pathologists, faced with the histological complexity of human clinical germ cell tumors, and the need to make decisions on patient treatment, have ceased to accept this simple terminology and the WHO recommends the term “mixed germ cell tumors containing embryonal carcinoma and teratoma”.^[^ ^16^ ^]^ Since the experimental work with human EC, and later ES cell lines was largely built upon earlier work in the laboratory mouse, we have suggested retention of the same terminology for the human tumors in a research setting.^[^ ^17^, ^18^ ^]^ More detailed accounts of the histological complexities of these tumors and the development of the terminology are provided in reviews by Damjanov and Solter,^[^ ^14^ ^]^ Damjanov,^[^ ^15^ ^]^ and Damjanov and Andrews.^[^ ^18^ ^]^

**The Knowles and Solter Group at the Wistar Institute**

Founded in 1892, with links to the University of Pennsylvania, the Wistar Institute in the 1960s and 1970s, under the leadership of Hilary Koprowski, had become a center for virology, immunology, and cancer cell biology. Research there led to new vaccines for rabies and rubella, and Peter Doherty's research into MHC restriction of the immune response earned him the Nobel Prize in 1996. In 1973, Barbara Knowles, who had joined the Institute in 1967, and Davor Solter, who had joined in 1973, started working together when building work forced their labs to move to an offsite annex for 2 years. At that time Barbara was using somatic cell hybrids to pursue her interests in the immune recognition and definition of human‐cell surface and tumor‐specific antigens^[^ ^19^, ^20^, ^21^ ^]^ while Davor, whose early work had contributed to understanding the relationship of teratomas to the mouse embryo,^[^ ^22^ ^]^ was continuing work on embryo culture in vitro,^[^ ^23^ ^]^ and on the pluripotent cells of mouse embryos. Drawing on their different interests and expertise, they developed a rapid immunosurgical procedure to isolate the ICM, by lysing the outer trophoblast cells for subsequent study^[^ ^24^ ^]^ and discussed the newly reported “F9 antigen”^[^ ^1^ ^]^ and the rather tentative methodology on which these findings were based.^[^ ^3^ ^]^ With Barbara's interests in the immunology of cell‐surface antigens they used the newly developed “hybridoma” technique^[^ ^25^ ^]^ to use monoclonal antibodies to define embryonic antigens like the “F9 antigen,” but not confused by the diverse reactivity found in conventional antisera. That work led to the definition of Stage Specific Embryonic Antigen‐1 (SSEA1)^[^ ^5^ ^]^ and several other SSEA's in subsequent studies.^[^ ^26^, ^27^ ^]^ Together they set out to characterize the cell surface molecules bearing these antigenic determinants.^[^ ^28^, ^29^, ^30^ ^]^ With pathologist Ivan Damjanov, they explored whether these antigenic determinants were expressed on cells of other mouse and human tissues including human teratocarcinomas.^[^ ^31^, ^32^, ^33^ ^]^

## 2 SEARCHING FOR A PLURIPOTENT HUMAN EC CELL LINE

A fortunate coincidence for our work with the human teratocarcinoma cells was that Ivan Damjanov, a clinical pathologist, and close friend and colleague of Davor and Barbara, was working across Philadelphia at Hahnemann Hospital. Ivan and Davor had previously worked together in Croatia on the biology of mouse teratocarcinomas, showing that they could be produced from ectopically transplanted embryos.^[^ ^22^ ^]^ Now as a practicing clinical pathologist Ivan had a strong interest in human germ cell tumors. One of our initial approaches to characterizing our teratocarcinoma cell lines was to see if they would produce xenograft tumors in immunodeficient, athymic “nude” mice. Several did, and Ivan was able to give his expert opinion that almost all of them were entirely composed of undifferentiated EC cells, disappointingly with no hint of differentiation. After some extensive comparison of the different cell lines we were led to the hypothesis that, contrary to our initial ideas, human EC cells do not express SSEA1.^[^ ^34^ ^]^ Further, since, we could occasionally detect low levels of HCG in the culture medium, a marker indicative of trophoblastic differentiation and widely used as a serum marker in germ cell tumor patients, we suggested that human EC cells might represent an earlier stage of embryogenesis than mouse EC cells, so providing a rationale for their lack of SSEA1; mouse EC cells were thought to represent inner cell mass cells that had passed the point of being able to generate trophoectoderm.

The next key step in the development of our ideas about human EC cells was that Barbara and Davor, working with Lynne Shevinsky, a graduate student who had previously been a technician with Karen Artzt and Dorothea Bennett at the Sloan‐Kettering Institute, identified another mouse embryonic antigen, SSEA3. SSEA3 is expressed by cleavage stage mouse embryo although not by the inner cell mass or mouse EC cells.^[^ ^26^ ^]^ Considering our thought that human EC cells represent an earlier stage of development to mouse EC cells, we wondered whether they might express SSEA3, while not expressing SSEA1. Indeed, Ivan together with Barbara and Davor found that in clinical samples of testicular germ cell tumors, the EC cells did express SSEA3 but not SSEA1.^[^ ^33^ ^]^

At the same time, I had extended our studies of the teratocarcinoma cell lines by single cell cloning of one line, 2102Ep, and found that these clones all formed pure ECs in xenografts in nude mice.^[^ ^35^ ^]^ Further, I discovered that if they were maintained at high cell density, the cells retained a morphology typical of EC cells, namely tightly packed with high nuclear to cytoplasmic ratios, and also expressed SSEA3, but not SSEA1. In contrast, if these same cells were passaged at very low densities, then many of the cells showed marked morphological changes while down regulating SSEA3 and turning on expression of SSEA1. In further studies, it seemed likely that at least some of these seemingly differentiated, SSEA1‐positive cells represented trophoblastic cells.^[^ ^36^ ^]^

Having made progress in defining human EC cells, it was a disappointment that none of the cell lines in our panel seemed capable of somatic differentiation, either in vitro, or in xenografted tumors. At this point serendipity came to the rescue. One of the teratocarcinoma cell lines in our collection was TERA2. It had been derived by Jørgen Fogh in the Sloan Kettering Institute from a lung metastasis of a testicular teratocarcinoma quite a few years before.^[^ ^37^ ^]^ However, in our hands it was one line that did not seem to display the characteristics that we had been using to define human EC cells.^[^ ^34^ ^]^ Its morphology was quite unlike that of other EC cells, it did not express SSEA3 and did not make tumors in nude mice; I was inclined to view that this cell line was composed of differentiated cells and not EC cells. However, by chance, a technician, Adrienne Mihalic, working with me took it into her head to inject a single nude mouse with TERA2 cells that she was growing while I was away on vacation. At that time the nude mice in our colony were not surviving very long, but this particular mouse did and I was astonished to see, contrary to past experience, that it developed a tumor, which I removed 81 days after injection. Fortunately, we put some of the tumor into culture, naming it NTERA2 (to indicate its passage through a nude mouse), while the remainder of the tumor was fixed and embedded. I remember taking the slides from this tumor to Ivan, and how animated and excited he became after looking at it – he said it was *undoubtedly* a teratocarcinoma with differentiated elements as well as nests of EC cells. Back in our lab, I was surprised that the cell line growing out, NTERA2, did not resemble the TERA2 line as we had been culturing it and, on testing, it expressed SSEA3 and *not* SSEA1. At that time, it was widely agreed that proof of pluripotency demanded evidence that differentiation occurred from clonal lines of undifferentiated cells to exclude the possibility that test cultures were in fact co‐cultures of presumed stem cells and unrelated differentiated cells, a test that Kleinsmith and Pierce had applied to the development of mouse teratocarcinomas^[^ ^13^ ^]^ and that Martin and Evans had applied to the identification of pluripotent mouse EC cells.^[^ ^38^ ^]^ I therefore established clonal sublines of NTERA2 from single cells isolated manually by micropipetting. After injection into new nude mice, these clonal lines all replicated the extensive differentiation seen in the original tumor, confirming their pluripotency.^[^ ^39^ ^]^

These observations posed an immediate conundrum – was the tumor really derived from TERA2, or had another line been mistakenly injected into the mouse? If the latter, a mix up with TERA1 seemed to be the most likely since those cells did resemble other human EC cell lines in morphology, growth patterns, and marker expression, though until then they too had failed to produce xenograft tumors. At that time, 1982, genetic finger printing had yet to be developed: the best approach then for confirming the identity of a cell line was to look at the expression patterns of allelic isozyme variants.^[^ ^40^ ^]^ Accordingly, I sent samples of NTERA2 to Jørgen Fogh at the Sloan Kettering Institute where he carried out isozyme analysis and compared the pattern with TERA1. The result was clear: NTERA2 was indeed derived from TERA2 and there had been no mix up. How to explain this? Our hypothesis was that perhaps we had not been culturing the cells correctly and that although there may have been EC cells present initially, in our hands most of these had been lost by differentiation, but that passage through the mouse had somehow rescued a few remaining but undetected EC cells. Indeed, by now, my experience with 2102Ep and other lines indicated that I should maintain these cultures permanently at high cell densities, while on reflection I had passaged TERA2 at relatively low densities. To test this hypothesis further, we obtained a new low passage culture of TERA2 from Jørgen Fogh and maintained it at high cell densities, when it was apparent that by morphology and marker expression it closely resembled the NTERA2 line and the other human EC cell lines in our panel. Further, when injected into nude mice, cells from this low passage TERA2 culture, as well as clones derived from it, also regularly continued to form well differentiated teratocarcinomas.^[^ ^41^ ^]^

Up until I isolated the NTERA2 line, I had been trying various known inducers of mouse EC cell differentiation to see whether they would induce differentiation of the seemingly nullipotent EC cell lines in our panel. Among these, I particularly focused on retinoic acid, a well‐established effective inducer of differentiation in mouse EC cell lines that were otherwise seemingly nullipotent.^[^ ^42^ ^]^ Retinoic acid had no morphologic effect on any of our lines^[^ ^43^ ^]^ until I tested it on NTERA2 cells. There was a certain excitement after looking at cultures of NTERA2 exposed to retinoic acid and seeing the dramatic change in cell morphology and the appearance of some cells that looked like neurons – something that I subsequently spent time confirming by looking for expression of appropriate markers.^[^ ^44^ ^]^ I mentioned this to a colleague in the University of Oxford, Chris Graham, who was also interested in trying to identify pluripotent human EC cell lines, and also to that point without great success. When he tested TERA2 he found essentially the same results that I had.^[^ ^45^ ^]^ Soon after, Martin Pera, then working in London, described the patterns of differentiation in another human pluripotent EC cell line, GCT27.^[^ ^46^, ^47^ ^]^

## 3 CELL SURFACE ANTIGEN MARKERS

During the 1960s and 1970s, there was a prevailing interest in the notion that specific cell:cell recognition played a key role in embryonic development, stemming in part from Moscona's studies of cell reaggregation in sponges^[^ ^48^ ^]^ and in part stimulated by studies of the immune system.^[^ ^49^ ^]^ Among some mouse developmental biologists, ideas about the mechanism of action of the T‐locus, summarized by Erickson^[^ ^3^ ^]^ also played a part. The structural complexity of cell surface associated carbohydrates also provoked the thought, exemplified by a UCLA Keystone Conference held in 1977,^[^ ^50^ ^]^ that these molecules had the capacity to provide a mechanism for specific cell:cell recognition, which was expected to play a central role in developmental processes. Indeed, it had become evident that mouse embryos and EC cells express high molecular weight carbohydrate structures that show marked developmental regulation during cell differentiation and that the “F9 antigen” of mouse EC cells is likely associated with these.^[^ ^51^, ^52^ ^]^

**FIGURE 1:**
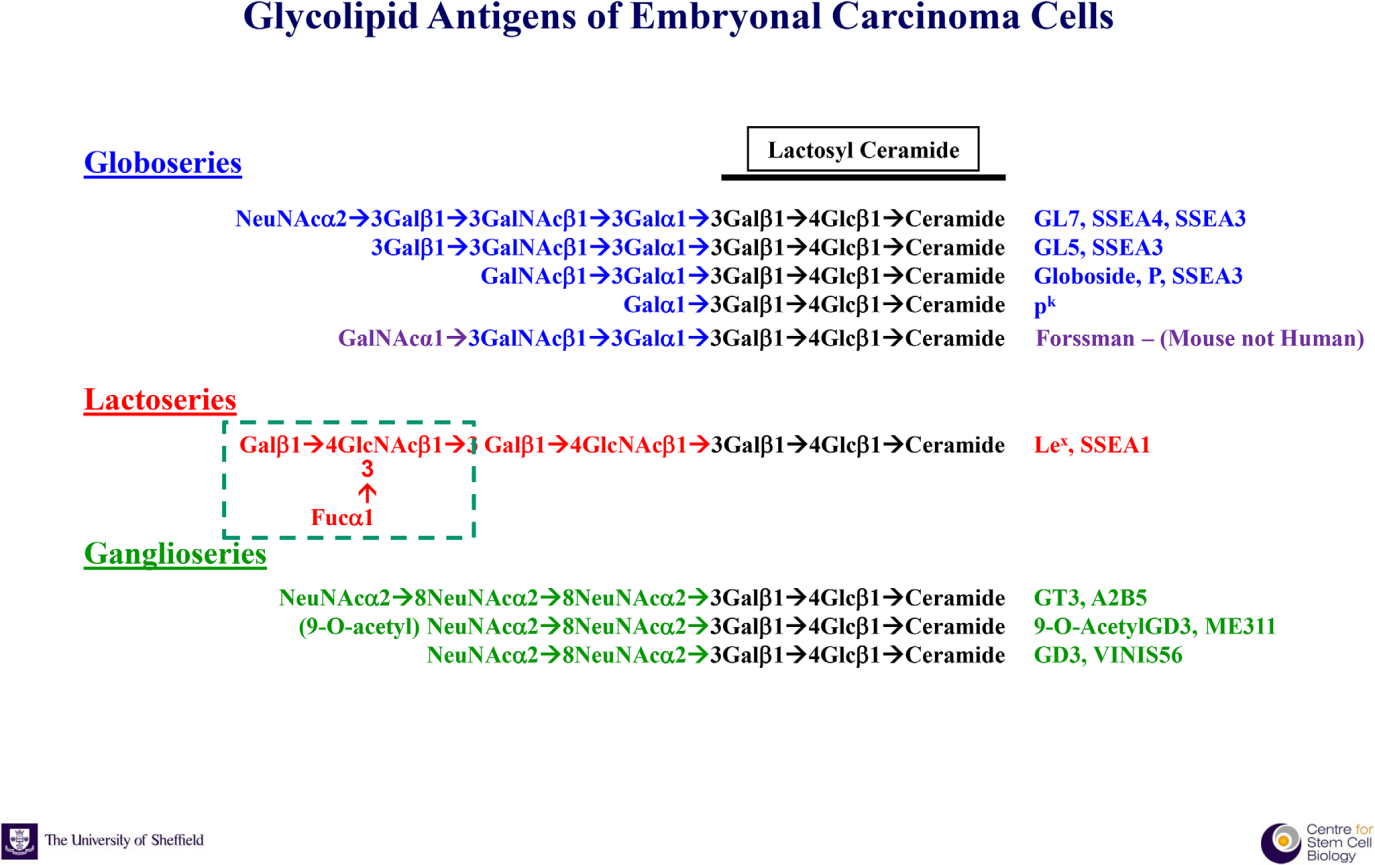
Glycosphingolipid antigens of human and mouse embryonal carcinoma (EC) cells. The glycolipid associated cell surface antigens that we identified in human EC cells and in their early differentiated derivatives are all glycosphingolipids that are synthesized from a common precursor, lactosyl ceramide. The first addition of galactose in an α1‐3 linkage initiates the synthesis of a series of globoseries structures that form the SSEA3 and SSEA4 epitopes characteristic of human EC cells: SSEA4 depends on the terminal sialic acid moiety, while the SSEA3 epitope does not require this.^[^ ^27^ ^]^ These same structures are also found on red blood cells where the tetrasaccharide, globoside, was identified as the P antigen; rare individual with the pp and p^k^ red blood cell phenotypes cannot extend the chain beyond lactosyl ceramide or gal(α1‐3)lactosyl ceramide, respectively.^[^ ^61^, ^62^ ^]^ The Forssman antigen, which is expressed on mouse EC cells, does not appear on human cells because they lack the enzyme to add *N*‐acetyl galactosamine to globoside.^[^ ^60^ ^]^ Differentiation of human EC cells like NTERA2 is associated with changes in the activity of the glycosyl transferases that extend the lactosyl ceramide precursor, resulting in downregulation of the synthesis of globoseries structures, and upregulation of lactoseries and ganglioseries structures that form the epitopes of various antigens recognized on the differentiated cells.^[^ ^57^, ^58^ ^]^ The SSEA1 or Le^X^ epitope is formed by fucosylation of the type 2 lactoseries oligosaccharide as shown.^[^ ^28^, ^29^ ^]^ The ganglioseries structures shown are those characterized during NTERA2 differentiation.^[^ ^57^, ^74^ ^]^

Against this background, our work also showed that the epitope defined as SSEA1 is a carbohydrate associated with high molecular weight glycoproteins.^[^ ^8^ ^]^ Collaborations of Knowles and Solter separately with the groups of Feizi^[^ ^28^ ^]^ and Hakomori et al.^[^ ^29^ ^]^ provided a clear identification of the SSEA1 epitope as *galactose(β1‐4)[fucosyl(α1‐3)]N‐acetyl‐glucosamine*, otherwise known as Lewis‐X (Le^X^), associated with an extended type 2 polylactosamine chain (Figure 1), which could be carried as a glycoprotein as well as a glycolipid.^[^ ^8^, ^53^ ^]^ Further, evidence that this carbohydrate structure does play a role in cell:cell interactions came from observations that monovalent oligosaccharides carrying the SSEA1/Le^X^ structure would cause disaggregation of blastomeres at the morula stage of mouse embryo development.^[^ ^54^, ^55^ ^]^ In this regard, Hakomori pointed out that the Le^X^ oligosaccharide itself is capable of homophilic aggregation, which could provide a mechanism for the homophilic aggregation of cells expressing SSEA1.^[^ ^56^ ^]^

Soon after the characterization of the SSEA1 epitope, further collaboration with Hakomori's group showed that SSEA3, and a related antigen, SSEA4, defined by another, new monoclonal antibody produced against the 2102Ep human EC cell line, were both carried as glycolipids, though associated with a globoside oligosaccharide backbone (Figure 1).^[^ ^27^ ^]^ Studying the developmental regulation of the SSEA1, SSEA3, and SSEA4 structures during NTERA2 differentiation with Bruce Fenderson, a postdoc in the Hakomori group, we found marked shifts in synthesis of the core structures of these antigens, with a switch from globo‐ to lactoseries or ganglioseries structures with different antigenic configurations marking different lineages.^[^ ^57^ ^]^ For example, the neural lineage cells were marked by the disappearance of globo‐ and lactoseries antigens and the appearance of gangliosides. These switches appeared to depend on changes in expression of several key glycosyl transferases, particularly rate limiting enzymes controlling addition of a third sugar moiety to the common precursor, lactosyl ceramide, to specify its extension into either globo‐, lacto‐, or ganglio‐series core structures^[^ ^58^ ^]^ (Figure 1). Further glycosylation of these core structure generated the different antigenic markers that we recognized with the available antibodies.

Despite a few suggestions that the SSEA1/Le^X^ structure might have functional activity, the significance of these carbohydrate structures, dependent upon so many developmentally regulated genes encoding a wide array of glycosyl transferases, was and remains obscure. Intriguingly, although mouse EC cells do not express SSEA3 or SSEA4, they do express another globoseries structure, the Forssman antigen.^[^ ^59^ ^]^ The Forssman antigen depends on the addition of a particular sugar to the globoseries core, but since humans lack the enzyme to accomplish that addition Forssman is not expressed on human cells.^[^ ^60^ ^]^ It maybe that it is the core structure of the oligosaccharides that is important rather than terminal specificities. However, SSEA3 and SSEA4 share antigenic determinants with those of the human P‐red blood group system.^[^ ^61^ ^]^ Although most individuals express these antigens they are not found on the red cells of a very rare group of people with the pp or p^k^ phenotypes. These individuals lack the enzymes to synthesize the globoseries core structure but show no developmental abnormalities^[^ ^62^ ^]^ suggesting that globoseries glycolipids are not required for normal development. Interestingly, women with these phenotypes do exhibit high rates of spontaneous abortion, perhaps due to immunological reactions against embryonic antigens such as SSEA3 and SSEA4. Further evidence for the lack of functional importance of these structures came from studies by Fenderson et al., who showed that Medaka fish embryos developed normally even when glycolipid synthesis was prevented by an inhibitor, PDMP.^[^ ^63^ ^]^ In the case of NTERA2 EC cells, culture in PDMP also failed to prevent their differentiation,^[^ ^64^ ^]^ while some other clonal EC cell lines derived directly from early passage TERA2 cells still exhibited pluripotency although they did not express SSEA3 or SSEA4.^[^ ^41^ ^]^ Curiously, though, the SSEA3 and SSEA4 negative TERA2 cells became permanently SSEA3 and SSEA4 positive after passage as a xenograft tumor in nude mice, suggesting that in some way expression of these antigens is a requirement for, or is somehow mechanistically linked to, tumor growth. Nevertheless, overall these observations have left a conundrum that is still not answered: why have organisms invested heavily in the extensive network of genes required to synthesize a wide array of exquisitely developmentally controlled complex carbohydrate structures that have no obvious essential function in embryonic development?

To expand the range of markers that could be used to follow EC cell differentiation, I produced additional hybridomas against undifferentiated EC cells. Among these, two, TRA‐2‐49 and TRA‐2‐54, were found to recognize the tissue non‐specific form of alkaline phosphatase, which is highly expressed on the surface of human EC cells, and is strongly downregulated upon differentiation.^[^ ^65^ ^]^ Two other monoclonal antibodies, TRA‐1‐60 and TRA‐1‐81, were found to recognize apparently related high molecular weight glycoproteins,^[^ ^66^ ^]^ but it seemed likely that the epitopes of both are also carbohydrate structures. Their expression is strongly downregulated during the differentiation of NTERA2 cells. Others also found similar high molecular weight glycoprotein associated antigens expressed by human EC cells.^[^ ^46^, ^67^ ^]^ The exact nature of the epitopes in each of these cases remains controversial. Initially they all appear to represent different modifications of keratan sulfate, a sulfated type 2 polylactosamine structure linked to a core protein^[^ ^68^ ^]^ but recent work has suggested the epitopes involve a hybrid type1/type2 lactosamine.^[^ ^69^, ^70^ ^]^ (Type 1 and type 2 lactosamine differ in the *Gal‐GlcNAc* linkage – *Gal(β1‐3)GlcNAc* in Type 1 and *Gal(β1‐4)GlcNAc* in Type 2). The nature of the core protein has also remained controversial, though it has been suggested to be podocalyxyn.^[^ ^71^ ^]^

Presaging the future larger scale collaborations of the International Stem Cell Initiative (ISCI) (Box 3) to agree standards for human ES cell research, a NATO Advanced Study Workshop held in Oxford in 1992 brought together many of the researchers then working on human teratocarcinomas to compare the many different EC cell lines that were then being studied by different groups.^[^ ^72^, ^73^ ^]^ That workshop concluded with general agreement that human EC cells were marked by expression of the globoseries glycolipid antigens SSEA3 and SSEA4, but not SSEA1, and the family of high molecular weight glycoproteins, typified by TRA‐1‐60, TRA‐1‐81, and GCTM2.

## 4 THE TRANSITION FROM EC TO ES CELLS

Soon after we began studies of human EC cells in the Wistar Institute, Martin Evans and Gail Martin, each independently, used their experience with mouse EC cells to derive phenotypically similar mouse embryonic stem (ES) cells directly from explanted mouse blastocysts,^[^ ^75^, ^76^ ^]^ to eliminate the possibility of spurious results that might arise because of the tumor adaptations of EC cells. Consequently, we and others began to explore the possibility of deriving human ES cells directly from human embryos. Only 3 years before, the first human baby had been born by the use of in vitro fertilization techniques to address problems of infertility^[^ ^77^ ^]^ opening access to human embryos for experimental purposes. However, there were widespread ethical concerns about the use of human embryos in any research so obtaining human ES cells remained notional for many years.

In the same general time frame, Jamie Thomson, a DVM/PhD student at the University of Pennsylvania who had done his thesis work in Wistar with Davor on the development of chimeras of androgenetic or gynogenetic embryos with normal mouse embryos and was well aware of our work with human EC cells, was doing his postdoctoral professional training at the Oregon Primate Center and at the University of Wisconsin where he had access to primate embryos. There he was able to derive ES cell lines from both rhesus and marmoset embryos.^[^ ^78^, ^79^ ^]^ From that experience with monkey ES cells and through a collaboration with Joseph Itskowtiz from Technion, in Israel, which gave him access to human embryos, Jamie was then able to derive the first ES cell lines from human embryos.^[^ ^80^ ^]^ Soon after, Pera and coworkers, again drawing on his experience with human EC cells, also described the derivation of human ES cells with similar characteristics.^[^ ^81^ ^]^ It was then not long before Yamanaka and coworkers, drawing on his studies of the molecular control of pluripotency, showed that it was possible to reprogram both mouse and human somatic cells to a state that closely resembled ES cells.^[^ ^82^, ^83^ ^]^ These cells he termed induced pluripotent stem (iPS) cells. About the same time, Thomson and coworkers used a slightly different set of reprogramming factors to also derive human iPS cells.^[^ ^84^ ^]^ It was with some satisfaction that these human ES and iPS cells were found to differ from the corresponding mouse cells and to express the same patterns of surface antigen markers that we had described for human EC cells.

Jamie had stayed in touch with me following our time together at the Wistar Institute, and soon after publishing his derivation of the ES cells he made them available to me, by which time I had moved to the University of Sheffield. Frustrated with the repeated requests and lack of success of others to cultivate his human ES cells in their own laboratories, Jamie also contacted Barbara Knowles, who had by then moved to The Jackson Laboratory in Bar Habor, Maine, and asked whether they could together establish a training course to teach newcomers how to culture and characterize these new human ES cells at The Jackson Laboratory. (This as a homage to Leroy Stevens who had made the seminal teratocarcinoma observations at the “JAX”). The Jackson Laboratory was in the throes of building a new Resources Building and having just designed a new training laboratory with ample space for just such an endeavor Barbara was shortly able to obtain NIH funding to support this effort. Co‐incidentally, healthcare funding agencies around the world had recognized the potential and also the difficulties of work with human ES cells and had formed a group, the International Stem Cell Forum (ISCF) (https://stemcellforum.org/) to help co‐ordinate ES cell research. Following a meeting in London in 2003, the ISCF asked me to lead an international research consortium, which became the ISCI (Box 3), to address concerns about standards of working with human ES cells. As a result, the ISCI organized a series of research meetings that coincided with the training course that Barbara and Jamie established at the Jackson Laboratory. Over the succeeding years these meetings and training courses, coupled with several research projects run by the ISCI consortium, provided a forum for the then relatively small, international research community, focused on human ES cells, to exchange ideas and develop standard approaches to working with ES cells, building on our previous experience with human EC cells.

**The International Stem Cell Initiative**

The International Stem Cell Initiative (ISCI) was formed and funded by the International Stem Cell Forum (ISCF) (https://stemcellforum.org/) following a scientific meeting held in London, in May 2023. Organized by a steering group comprising Nissim Benvenisty (Israel), Ron McKay (USA), Martin Pera (Australia), Janet Rossant (Canada), Henrik Semb (Sweden), and Glyn Stacey (UK), and chaired by Peter Andrews (UK), the ISCI was a consortium of research groups from around the world that had being deriving ES cells following the initial publications of Jamie Thomson in 1998 and Martin Pera in 2000.^[^ ^80^, ^81^ ^]^ It initially sought to compare the different embryonic stem (ES) cell lines that had been described up to that time and establish commonly agreed criteria for their characterization. It also organized workshops to plan research projects and discuss results, several held in conjunction with an ES cell training course held at the Jackson Laboratory in Bar Harbor and run by Jamie Thomson and Barbara Knowles. Following its first study,^[^ ^85^ ^]^ the ISCI took advantage of the opportunity for collaboration between many of the prominent research groups studying human ES, and later also iPS cells, to compare media that were used for their culture,^[^ ^86^ ^]^ to study their propensity for genetic change on prolonged culture, and the implications of genetic variants for their application in research and medicine^[^ ^87^, ^88^, ^89^ ^]^ and to assess their propensity for differentiation.^[^ ^90^ ^]^

## 5 LESSONS LEARNED

Like much of basic science, our work in the Wistar Institute to characterize human EC cells was driven by circumstance and serendipity, rather than a grand design. In part, the circumstance was provided by the efforts embryologists to harness the biology of teratocarcinomas to address problems of embryonic development in the mouse.^[^ ^6^ ^]^ If they were useful for understanding the mouse embryo, why not use human teratocarcinomas to explore human development? Surprisingly, though, funding such research proved initially difficult, with reviewers asking why work with a human system when the mouse was available – an ironic view given the differences between mouse and human ES cells discovered subsequently and the potential for human ES cells to address problems in human health. But it was then chance events and observations that allowed us to progress, like the survival of a single mouse injected with TERA2 cells that led us to discover and characterize the properties of pluripotent human EC cells.

**FIGURE 2:**
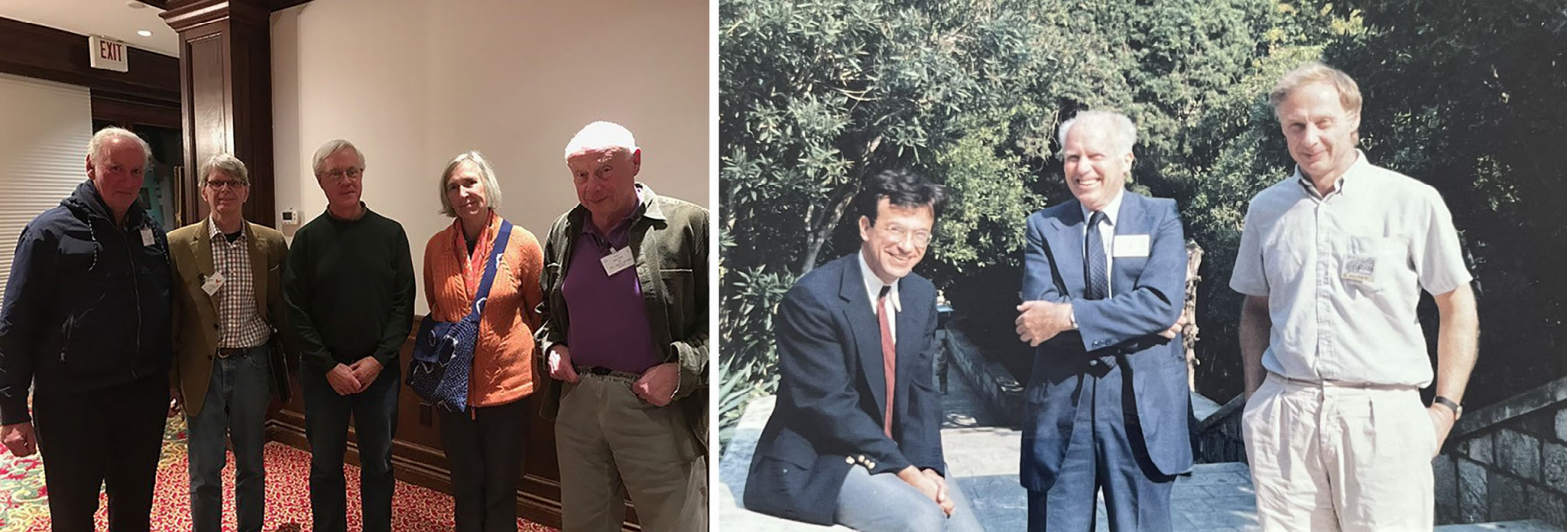
*Dramatis personae*: Some key players in the development of pluripotent human embryonal carcinoma (EC) cell lines. (A) Christopher Graham (left) and Barbara Knowles and Davor Solter (Right) with John Gearhart (second from left) and Joe Nadeau (third from left) at a symposium to honor the work and life of Leroy Stevens, held at the Jackson Laboratory October 14–16, 2016.^[^ ^96^ ^]^ (B) Ivan Damjanov (left) and Davor Solter (right) with Prof. Nikola Skreb (center) on the occasion of his retirement in 1986.

For me personally, the critical circumstance was the opportunity to work in a lab led by two people with broad expertise and critical insight who could provide support, yet allow me the freedom to “play” and explore my own ideas. The opportunities for team work, discussion, and collaboration between small groups of researchers with diverse experience and interests was essential. Within Wistar, and nearby, Barbara Knowles’ expertise in cancer cell biology and immunology, Davor Solter's in mouse embryology, and Ivan Damjanov's in clinical pathology provided essential insights (Figure 2). Further afield it was interactions with a wider community with interests in human germ cell tumors that drove development of the field. A NATO international research collaboration grant allowed me to carry out projects with my colleague, Peter Goodfellow, who had moved to the ICRF in London. Not only did this help with; genetic analysis of some of the antigens but also brought me into contact with other groups in the UK, notably Martin Pera, then at the Ludwig Institute, and Chris Graham in Oxford, both of whom were pursuing studies of human EC cells, also presaging the eventual development of human ES cells.^[^ ^91^ ^]^ Meetings between these groups fostered new ideas and allowed us to confirm our findings.

## 6 CONCLUSION

As for mouse ES cells, studies of human EC cells from germ cell tumors paved the way for the isolation and characterization of human ES cells, and later iPS cells. An essential element in this development was the free collaboration between researchers worldwide following their own curiosity and interests. These cells now offer extensive opportunities for applications in human healthcare, as discussed by other authors in this issue of BioEssays. For example, the original intention to use human teratocarconoma‐derived cell lines as discovery tools for human embryogenesis is now coming to pass as the use of synthetic embryos/gastruloids made from human ES/iPS cells promises mechanistic insights into early postimplantation human development in the foreseeable future.^[^ ^92^ ^]^ Meanwhile great strides have been made toward using these cells for studying disease processes, such as in the heart,^[^ ^93^ ^]^ or replacing diseased or damaged tissues in regenerative medicine, for example, retinal pigment cells in the eye, or dopaminergic neurons in the brains of patients with Parkinson's disease.^[^ ^94^, ^95^ ^]^ The journey from human EC to human ES and to human iPS cells has been crucial for this passage.

## CONFLICT OF INTEREST STATEMENT

The author receives Royalty Income from the Wistar Institute from non‐exclusive commercial licences to the TRA series of monoclonal antibodies. The author is also a member of the SAB of TreeFrog Therapeutics, and is a consultant to BlueRock Terapeutics LP.

## Data Availability

Data sharing is not applicable to this article as no new data were created or analyzed in this study.
